# Is percutaneous endoscopic lumbar discectomy necessary for learning the unilateral biportal endoscopy technique?

**DOI:** 10.3389/fsurg.2025.1530325

**Published:** 2025-04-11

**Authors:** Yiwei Xie, Yicheng Chen, Qifeng Yu, Yi Liu, Xin Gu, Xiaojian Ye

**Affiliations:** Department of Orthopedics, Tongren Hospital, Shanghai Jiao Tong University School of Medicine, Shanghai, China

**Keywords:** percutaneous endoscopic lumbar discectomy (PELD), unilateral biportal endoscopy (UBE), learning curve analysis, cumulative sum (CUSUM), surgical complications

## Abstract

**Objective:**

This study aims to investigate the effect of prior percutaneous endoscopic lumbar discectomy (PELD) surgical experience on the learning curve of the unilateral biportal endoscopy (UBE) technique.

**Methods:**

A total of 200 patients undergoing single-segment UBE surgery were enrolled. The procedures were performed by four surgeons, who were divided into two groups based on whether they had prior PELD experience (Group A: with; Group B: without). Proficiency in UBE technique was defined as a surgery time of less than 80 min. The cumulative sum analysis (CUSUM) method was used to analyze each surgeon's learning curve. Clinical efficacy was evaluated using patient-reported outcomes (PROs) after surgery: Modified Macnab, VAS-leg, VAS-back, and ODI scores. Follow-up information was obtained 12 months postoperatively.

**Results:**

The number of cases required for Group A surgeons to achieve proficiency were 17 and 18, significantly fewer than the 25 and 27 cases for Group B surgeons. No significant differences in clinical outcomes were observed between the two groups. The complication rates for Group A and Group B were 5 and 14, respectively.

**Conclusion:**

Prior PELD surgical experience facilitates learning the UBE technique. This experience further aids in shortening surgical times, lowering complication rates, and decreasing the need for reoperation.

## Introduction

In the past few decades, endoscopic spine surgery (ESS) has seen remarkable progress, with ongoing advancements in endoscopic instruments and surgical techniques aimed at optimizing efficacy, improving patient prognosis, reducing complications, and minimizing surgical damage (1, 2). Today's single-channel and biportal endoscopic spine techniques originate from arthroscopy technology (3). Osman SG reported a transforaminal lumbar interbody fusion using arthroscopy in 2012 (4), with approaches like single-portal, bilateral biportal, and unilateral biportal, marking early explorations of techniques such as unilateral biportal endoscopy (UBE), although no standard procedure was developed at that time. At that time, due to the growing popularity of transforaminal endoscopic techniques, biportal techniques were not widely emphasized or promoted. Percutaneous endoscopic lumbar discectomy (PELD), a form of single-port endoscopic spine technique, has achieved significant clinical success (5). However, the transforaminal endoscopic technique has gradually been found to have several limitations. In recent years, UBE technology has regained attention among spine surgeons (6).

Compared to PELD, the UBE technique positions the endoscope and instruments in two separate portals; its surgical view and mode are more similar to traditional open surgery, aligning with the surgeon's operational habits, enhancing surgical efficiency (7). As a result, UBE technology has been widely favored by spine surgeons (8–11). Previously, the choice of single or biportal endoscopy was mainly determined by the surgeon's preferences and experience. However, as interest in ESS has increased, many spine surgeons with no prior experience in endoscopic surgery have begun learning biportal endoscopy. UBE technology is a relatively simple starting point and can be described as a surgeon-friendly biportal endoscopic technique, as it involves more familiar procedures and more accessible instruments (9, 10). Nonetheless, no previous studies have investigated how prior PELD experience influences UBE learning. Therefore, the objective of this study is to address this gap in the literature by analyzing the learning curves of two groups of surgeons with varying levels of endoscopic surgical experience in learning the UBE technique, and to evaluate whether the learning curve impacts patient-reported outcomes after surgery (PROs). We used the cumulative sum (CUSUM) technique to evaluate the learning curve, as this method has been shown to detect subtle changes and is widely accepted for monitoring different stages of skill acquisition (12–15).

## Methods

### Study design

This retrospective study received approval from the Ethics Committee at Tongren Hospital, affiliated with Shanghai Jiao Tong University School of Medicine. Clinical information was continuously collected from patients who underwent single-segment UBE surgery between November 2020 and May 2021. The procedures were conducted by four doctors, and the inclusion criteria were as follows: (1) Patients with clearly defined indications for single-segment lumbar disc herniation and lumbar stenosis; (2) American Society of Anesthesiologists (ASA) classification of I–III; (3) Patients who completed at least 12 months of follow-up successfully and provided complete data. The exclusion criteria were as follows: (1) Patients with severe underlying diseases; (2) Patients with a previous history of spinal surgery; (3) Patients with lumbar instability, infections, or tumors; (4) Patients with other multisegmental lumbar diseases requiring intervention; (5) Surgeries performed by other surgeons. Two surgeons had prior experience with PELD (>100 cases) (Group A); The other two surgeons had no PELD experience (Group B). The 100 cases were defined based on the clinical expertise of experienced doctors in our department, with both groups having no prior UBE surgical experience. Based on the above inclusion and exclusion criteria, a total of 200 patients were included in this study.

### Surgical technique

**Figure 1:**
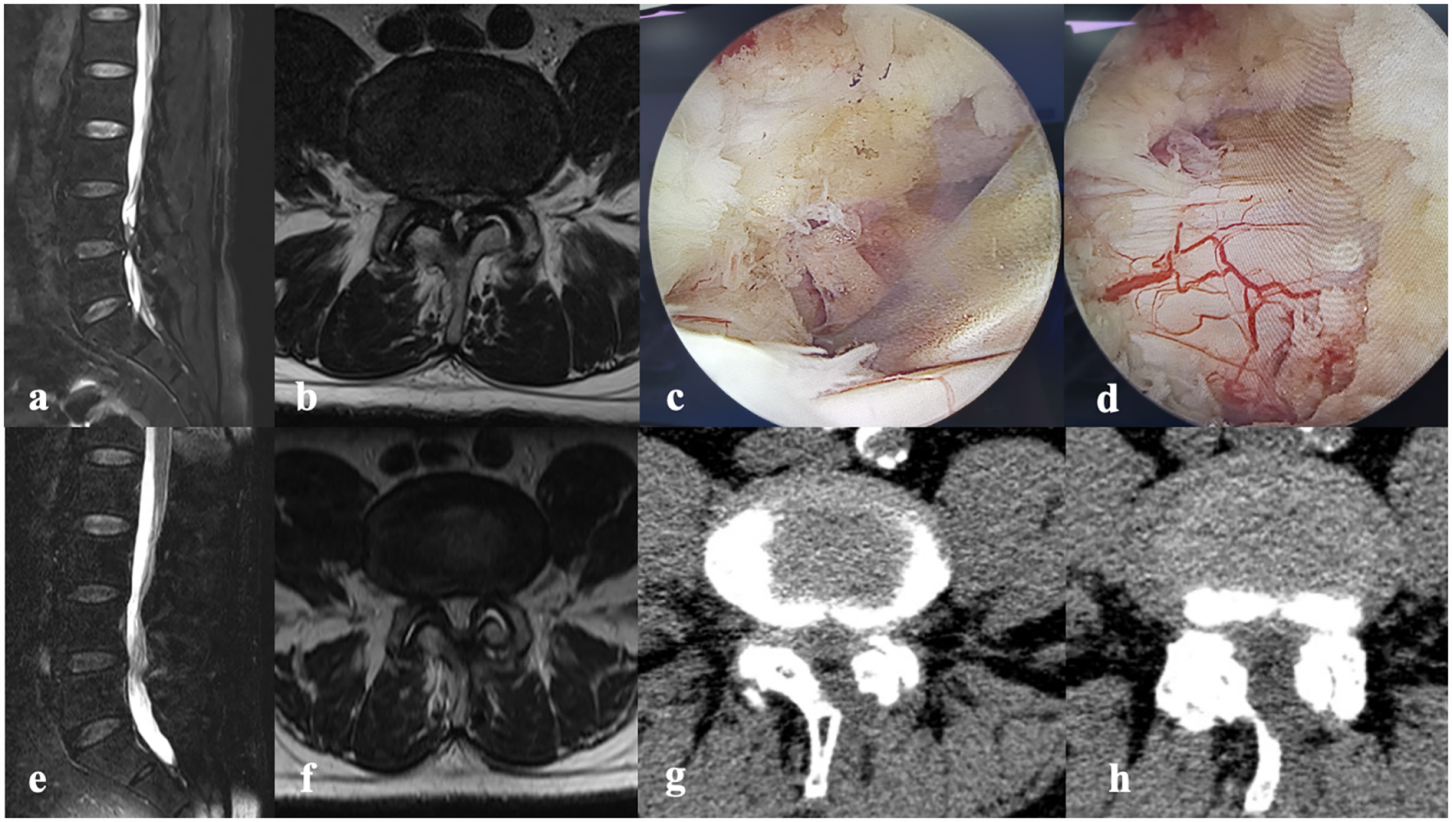
A 72-year-old male had numbness in both lower limbs accompanied by intermittent claudication for 2 years, with symptoms occurring after walking 200 m. No significant positive findings on physical examination. ODI score is 40. **(a)** The sagittal view of MRI. **(b)** The axial of MRI. **(c,d)** Relieved compression under endoscopy. **(e,f)** The sagittal/axial view of MRI showing the lumbar spine after surgery. **(g,h)** The axial view of CT.

UBE: The procedure is performed under general anesthesia, with the patient in a prone position. Under the monitoring of the C-arm lateral fluoroscopy, the surgical table is adjusted to make the target intervertebral space as vertical as possible to the horizontal plane. A skin incision is made at a point 1 cm laterally from the outer edge of the affected side's pedicle, with incisions made 1.5 cm above and below the intervertebral space, followed by the gradual insertion of a guide rod and an expansion tube, intersecting precisely at the level of the intervertebral space. A light source channel and a working channel are created, with the left-sided incision designated for observation and the right-sided incision designated for working. Sequential dilators are used to expand both channels, and the dilators can palpate the lower edge of the superior lamina and the interlaminar space. Using a plasma radiofrequency knife, structures such as the lower edge of the superior lamina, the root of the spinous process, the upper edge of the inferior lamina, the inner edge of the articular facet, and the ligamentum flavum are exposed as needed. Depending on the surgical requirements, instruments like powered drills or gun-type bone forceps are utilized to remove bone from the lower edge of the superior lamina, the upper edge of the inferior lamina, and the medial side of the facet joint. The ligamentum flavum is excised as required for decompression. The spinal canal is explored to identify and remove the intervertebral disc compressing the nerve. After confirming that there is no nerve compression or active bleeding, the instruments are withdrawn, and the incision is sutured. A typical case is shown in Figure 1.

### Data collection

Clinical data of patients were extracted from the hospital information system and electronic medical records. The following data were analyzed:

1. Baseline data: age, gender, BMI, disease duration, side of surgery (left or right), type of disease, surgical level.
2. Outcome evaluation: surgery duration, postoperative hospital days, surgical complications, and patient-reported outcomes (VAS-leg, VAS-back, ODI, modified Macnab score) at different preoperative and postoperative time points.

### Learning curve analysis

The learning curve (LC) was analyzed using the cumulative sum (CUSUM) method, with the following formula: $\mathrm{CUSUM}=\sum_{i=1}^{n}\left(X_{i}-u\right)$, where *X*_*i*_ represents the actual surgery time for each patient, and *u* represents the average surgery time for the group of patients. The CUSUM value is obtained by cumulatively summing the differences between the surgery time of each patient in chronological order and the average surgery time of the whole group. The learning curve is plotted with the case number on the *x*-axis and the CUSUM value on the *y*-axis.

### Statistical analysis

Statistical analyses were performed using SPSS 22.0 (IBM Corp., Armonk, NY, USA) and R language (version 4.4.0, R Foundation for Statistical Computing, Vienna, Austria). Continuous variables were expressed as means ± standard deviations (SD), while categorical variables were presented as numbers (*n*). Normality of data distribution was assessed using the Shapiro–Wilk test. To evaluate differences between groups, independent samples *t*-tests were used for normally distributed continuous variables, and the Mann–Whitney *U* test was applied for non-normally distributed data. For categorical variables, Fisher's exact test was used to compare proportions. Within-group differences (e.g., preoperative vs. postoperative measurements) were analyzed using paired *t*-tests for normally distributed data and the Wilcoxon signed-rank test for non-normally distributed data. The learning curve was plotted using R language, with the inflection point indicating the transition from the learning phase to the proficiency phase. A two-tailed *P*-value < 0.05 was considered statistically significant.

## Results

### Baseline characteristics between the two groups

Among all 200 enrolled patients, there were 106 males and 94 females. The mean age was 46.24 ± 9.51 years. All patients were followed up for at least 12 months. The demographic characteristics of the enrolled patients are shown in Table 1.

**Table 1:** Characteristics of the patients.

Characteristic	Group A	Group B	*P* value
Age	46.94 ± 10.09	45.54 ± 8.89	0.299
Gender (male/female)	49/51	57/43	0.257
BMI (kg/m^2^)	25.64 ± 1.40	25.89 ± 1.49	0.234
Disease duration (months)	16.25 ± 5.317	15.65 ± 4.96	0.410
Approach side (left/right)	48/52	41/59	0.319
Surgical segments			
L3/4	10	9	0.536
L4/5	74	68	
L5/S1	16	22	

BMI, body mass index.

### Perioperative data and follow-up results

Both groups successfully completed surgery, with the average operative time in Group A (with prior PELD experience) being 85.77 ± 21.93 min, significantly shorter than in Group B (without prior PELD experience) at 94.05 ± 19.68 min (*P* < 0.05). The difference in postoperative hospitalization time between the two groups was not statistically significant. Regarding postoperative complications, neither group experienced severe complications such as nerve injury, major vascular injury, or deep infection. In Group A, 4 patients had dural tears without significant neurological symptoms and were able to ambulate after 3 days of bed rest; 1 patient developed right leg pain 2 days post-surgery, and lumbar MRI revealed an epidural hematoma, which was resolved with conservative treatment. In Group B, 1 patient experienced postoperative lower limb pain, saddle anesthesia, and urinary difficulties, with lumbar MRI showing an epidural hematoma. Symptoms improved after UBE-guided hematoma evacuation. Another patient in Group B developed bilateral foot drop, with grade 1 strength in the tibialis anterior and extensor hallucis longus muscles. After conservative rehabilitation, muscle strength recovered to grade 4 within 3 months. 12 patients experienced dural tears, and 1 of them was converted to open surgery for repair.

The VAS scores at 2 weeks, 6 months, and 12 months postoperatively in both groups were significantly reduced compared to preoperative scores (*P* < 0.05), with no statistically significant difference between the two groups at each time point (*P* > 0.05). The ODI scores in both groups at 6 and 12 months post-surgery were significantly lower than preoperative scores (*P* < 0.05). There was no statistically significant difference in ODI scores between the two groups at any postoperative time point (*P* > 0.05). At the final follow-up, based on the modified MacNab criteria, Group A had 78 excellent cases, 18 good cases, 3 fair cases, and 1 poor case. In Group B, there were 73 excellent cases, 21 good cases, 3 fair cases, and 3 poor cases. No statistically significant differences in postoperative outcomes were observed between the two groups (*P* > 0.05) (Table 2).

**Table 2:** Comparison of intra-operative and post-operative data between two groups.

Items	Group A	Group B	*P* value
Operative time (minutes)	85.77 ± 21.93	94.05 ± 19.68	**0** **.** **005**
Postoperative hospital stays(days)	4.63 ± 1.14	4.56 ± 1.18	0.671
Complication (yes/no)	5/95	14/86	**0**.**030**
VAS (back)			
Pre-operative	6.65 ± 1.09	6.62 ± 1.25	0.856
2 weeks PO	1.74 ± 0.76	1.78 ± 0.69	0.680
6 months PO	1.19 ± 0.68	1.25 ± 0.68	0.500
12 months PO	1.29 ± 0.71	1.25 ± 0.58	0.663
VAS (leg)			
Pre-operative	6.23 ± 1.21	6.49 ± 1.22	0.132
2 weeks PO	1.82 ± 1.10	1.63 ± 1.03	0.211
6 months PO	1.35 ± 0.82	1.41 ± 0.87	0.185
12 months PO	1.22 ± 0.72	1.34 ± 0.73	0.242
ODI			
Preoperative	65.95 ± 8.59	67.57 ± 8.16	0.173
6 months PO	16.30 ± 6.98	14.62 ± 7.01	0.091
12 months PO	13.78 ± 7.15	14.11 ± 6.34	0.730
Modified Macnab criteria (excellent: good: fair: poor)			
12 months PO	78:18:3:1	73:21:3:3	0.706

PO, postoperative.

Bold values indicate statistically significant differences.

### Learning curve

**Figure 2:**
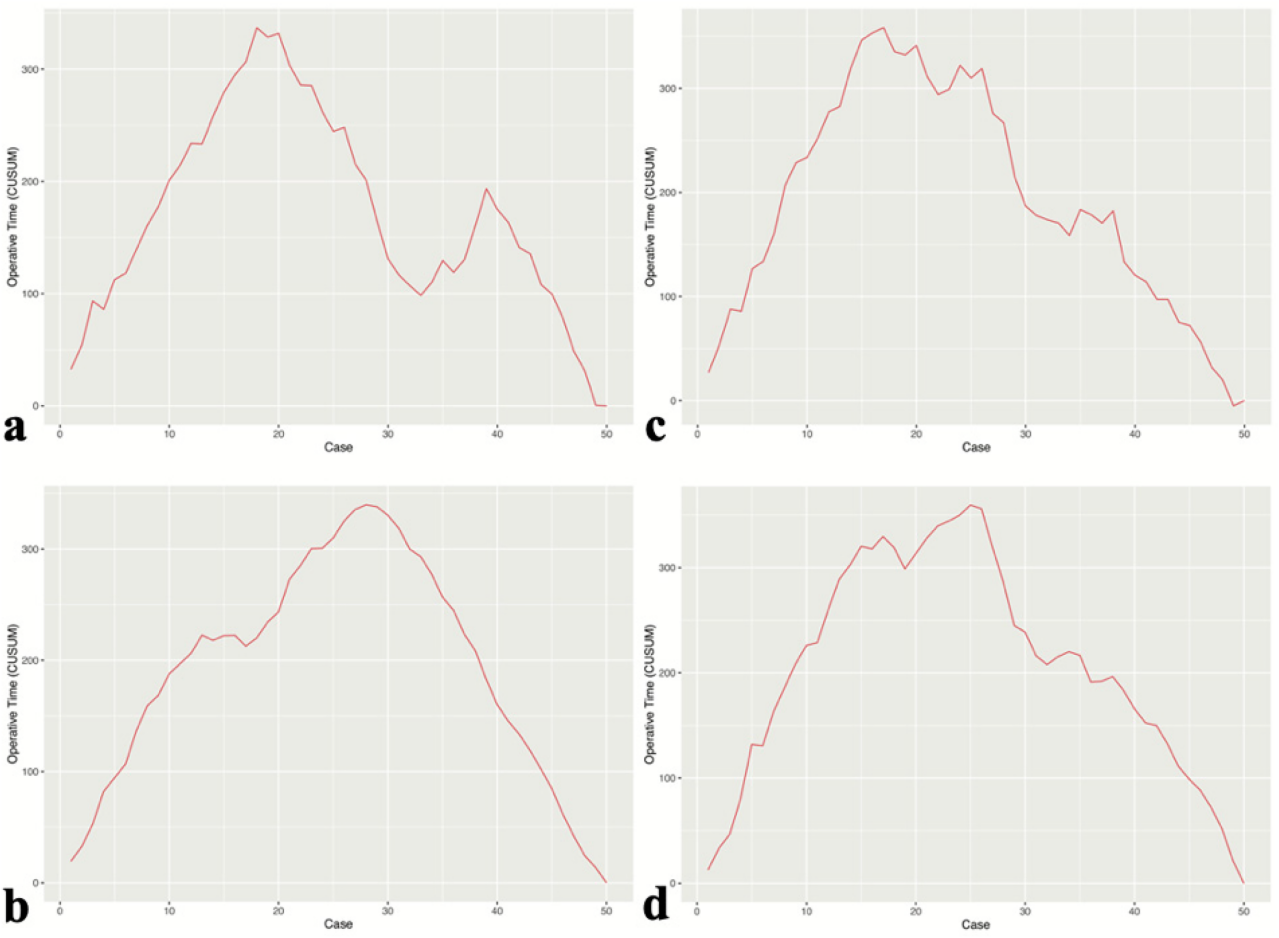
Learning curves of surgeons in group A **(a,b)** and group B **(c,d)**.

The learning process of each surgeon was demonstrated using the CUSUM learning curve. CUSUM analysis was used to define the case point at which proficiency in UBE surgery was achieved (Figure 2). Compared to Group B, surgeons in Group A required fewer cases to achieve proficiency in UBE technique (17 and 18 cases vs. 25 and 27 cases).

## Discussion

As minimally invasive surgical principles gained momentum and endoscopic technology advanced, single-portal endoscopy (PELD) and biportal endoscopy (UBE) have become key tools in spinal surgery. However, as PELD became widely adopted, clinicians began to realize its limitations in managing complex spinal disorders. Since both operation and visualization depend on a single channel, PELD faces challenges such as a narrow surgical field and limited instrument manipulation when dealing with complex lesions (e.g., lumbar spinal stenosis) (16). To address these limitations, biportal endoscopy emerged as a solution. In 1996, Italian scholar De Antoni first described the application of an arthroscopic system-assisted posterior spinal approach using two independent percutaneous portals in lumbar surgery with the patient in the lateral decubitus position (17). In 2017, Korean scholar Heo et al. were the first to propose the concept of unilateral biportal endoscopy (UBE) (18). It has only matured and seen widespread application in the last decade (18–20). UBE utilizes two independent channels for surgery: one for inserting the endoscope to observe the surgical area, and the other for operating instruments. This design significantly expands the surgical field and improves the flexibility and safety of the procedure. Biportal endoscopy has shown great advantages in treating complex spinal diseases, particularly lumbar spinal stenosis (21, 22). Compared to single-portal endoscopy, UBE allows surgeons to more easily expose and manage the affected area, especially during procedures such as nerve root decompression and facet joint resection. Additionally, since the surgical field and operative methods of UBE are closer to traditional open surgery, many surgeons who are accustomed to open surgery find it easier to accept this technique compared to PELD. In recent years, with continuous improvements in UBE technology and equipment, its application has gradually expanded and has replaced traditional open surgery in some complex procedures.

We acknowledge that the learning curve advantage of transitioning from PELD to UBE may seem intuitive; however, we believe that systematically demonstrating this phenomenon with objective data enhances its academic value. This study aimed to evaluate the impact of previous PELD surgical experience on the learning of UBE technology by comparing the learning curves and surgical outcomes of doctors with and without PELD experience, from which several key conclusions were drawn. First, the study demonstrated that PELD surgical experience significantly shortened the UBE learning curve. Specifically, doctors with PELD experience were able to reach proficiency in UBE technology more quickly. The data indicated that surgeons with PELD experience required less cases to reach proficiency, compared to those without such experience (17 and 18 cases vs. 25 and 27 cases). This result suggests that prior PELD experience makes it easier for doctors to adapt to and master biportal endoscopy techniques, reducing operative time and minimizing the risk of surgical failure. Secondly, although there was no significant difference in postoperative clinical outcomes between the two groups, doctors with PELD experience demonstrated better performance in terms of complication rates. This was particularly evident in serious complications such as nerve injuries and dural tears. Furthermore, although there were no significant differences between the two groups in terms of postoperative VAS scores, ODI scores, or Modified MacNab criteria, it is worth noting that Group A surgeons demonstrated higher levels of efficiency and precision in surgical operations. Surgeons with prior PELD experience exhibit significant advantages in these aspects. Both PELD and UBE are performed in an aqueous environment, which allows surgeons with PELD experience to become more familiar with the endoscopic field of view and achieve more precise intraoperative hemostasis. Additionally, the single-channel approach of PELD requires surgeons to perform delicate operations within a limited visual field. This skill is directly transferable to UBE, enabling PELD-experienced surgeons to adapt more quickly to the biportal system. Therefore, this study demonstrates that surgeons with prior PELD experience have overcome the steep initial learning curve of endoscopic surgery, allowing them to master UBE techniques more rapidly. The findings of this study hold significant clinical relevance. For doctors with PELD experience, the learning process of UBE technology is more efficient, suggesting that hospitals and training institutions can design more personalized training programs based on the doctor's previous surgical experience, thereby optimizing learning outcomes. In addition, this study provides empirical support for how UBE technology can be better promoted and applied in future clinical practice.

PELD and UBE, as key approaches in modern minimally invasive spine surgery, share many similarities in terms of surgical principles and techniques, but they also differ significantly in their specific applications and technical details. In terms of similarities: First, both PELD and UBE are spinal endoscopic surgeries, and both are performed in a water-based medium. The use of a water medium not only cools the surgical area, preventing thermal damage, but also helps flush and expand the surgical field to some extent. However, incomplete hemostasis during surgery can result in unclear visualization and difficulty identifying tissue structures. Additionally, both procedures aim to address spinal lesions through minimally invasive approaches, reducing damage to surrounding healthy tissues. Compared to traditional open surgery, PELD and UBE offer reduced trauma, quicker recovery, and fewer postoperative complications, resulting in shorter recovery times and significantly improved patient quality of life (11, 23). These advantages must be evaluated in the context of strict patient selection criteria and should not be generalized to all types of lumbar disc herniation and lumbar stenosis. Despite the aforementioned similarities, there are significant distinctions between PELD and UBE in terms of surgical operation methods and indications. PELD mainly relies on a single channel for both operation and visualization and is primarily used to treat lumbar disc herniation. Its surgical field and operational space are relatively limited, especially when addressing complex lesions, which may pose challenges. In contrast, UBE technology utilizes two independent channels, one for endoscopic visualization and the other for surgical operations. This setup offers a wider field of view and greater flexibility, enabling UBE to more effectively address complex spinal conditions, particularly lumbar spinal stenosis. The dual-channel system allows surgeons to achieve clearer visibility and handle lesions over a larger area. Also UBE facilitates the decompression of the contralateral side and enables the identification of both the traversing root and the contralateral facet, all while minimizing the resection of bone structure (24, 25). In a recent study, Liu et al. compared the efficacy of PELD and MED in treating foraminal and extraforaminal lumbar disc herniations. The results showed that while both surgical techniques demonstrated significant effectiveness over a 2-year follow-up period, PELD provided superior relief of low back pain. However, patient dissatisfaction was mainly associated with postoperative low back pain, surgical cost, and symptom recurrence, despite similar overall satisfaction rates between the two procedures (26). Additionally, another study comparing the efficacy and safety of UBE with other spine surgeries found that UBE was superior to MED in relieving back pain on the first postoperative day. However, one-day leg pain relief, long-term outcomes, and safety were comparable between UBE and MED. Similarly, UBE and PELD showed no significant differences in terms of short-term pain relief, long-term efficacy, and safety. Further evidence is needed to assess the efficacy and safety of UBE compared to PLIF. These findings suggest that UBE and PELD each have their advantages and limitations (27). Aspiring endoscopic surgeons can learn either the full-endoscopic or biportal technique first, depending on their preference or the circumstances of their surgical teams. Moreover, the choice of surgical approach should also be tailored to the specific conditions of the patient.

In this study, the primary complications associated with UBE technique included nerve injuries and dural tears. These complications were mainly attributed to inadequate hemostatic management, leading to increased intraoperative bleeding and subsequent subdural hematoma. Additionally, insufficient adaptation to endoscopic anatomy resulted in operative errors, causing dural tears. Furthermore, limited spatial awareness during endoscopic procedures increased the risk of misoperation, leading to nerve root injury. Notably, this study found that surgeons with PELD experience exhibited significant advantages in handling these complications. Specifically, Group A surgeons (those with PELD experience) benefited from the vast experience gained from single-portal endoscopic procedures. In PELD surgery, surgeons usually need to perform delicate operations in a relatively confined space, including accurate localization and excision, and effective control of intraoperative bleeding. Consequently, when transitioning to UBE, these surgeons could quickly adapt to the biportal setup and effectively apply endoscopic hemostatic techniques, maintaining clear visualization and reducing the incidence of nerve injuries and dural tears. In contrast, for doctors without PELD experience (Group B), while they may have gained some experience in traditional open surgery, they required a longer adaptation period when facing endoscopic surgery. This group had a higher complication rate, especially during the early learning phase, reflecting their challenges in orientation, precise manipulation, handling complex lesions, and controlling intraoperative bleeding under endoscopy. This suggests that doctors without PELD experience may require more training and practice when learning UBE technology, especially in endoscopic hemostasis and nerve protection, to reduce the risk of complications.

This study demonstrates that PELD experience not only helps shorten the learning curve for UBE technology but also has a significant advantage in reducing surgical complications. This finding highlights the transferability and cumulative effect of surgical experience, providing valuable insights into how systematic training could reduce endoscopic surgery complications in the future. In this era of rapid technological advancement, implementing augmented reality (AR) surgical navigation may help shortening the learning curve (28). One of the key advantages of AR) in clinical practice is its ability to intuitively display anatomical information. With AR, surgeons can directly visualize internal structures in real-time, which is especially valuable for minimally invasive spinal surgery. Conventional navigation systems require surgeons to shift focus to a separate screen, increasing cognitive load. AR eliminates this distraction by integrating critical information into the surgical field, enhancing efficiency, precision, and shortening the learning curve.

This study is a clinical retrospective study. Future research should further explore the impact of different surgical backgrounds on the learning curve, such as evaluating the adaptability of surgeons with only open surgery experience but no PELD experience in learning UBE. Additionally, with the advancement of AR technology, its application in endoscopic surgery is worth investigating. Future studies could assess how AR navigation can help shorten the learning curve for UBE techniques.

## Data Availability

The raw data supporting the conclusions of this article will be made available by the authors, without undue reservation.
